# Loot

**Published:** 1871-06

**Authors:** 


### Loot.

*Chloralum.*

The new antiseptic commended by Professor Gamgee, and known as chloralum bids fair to be of much value in its applications in domestic economy and in medicine. The advantages claimed are the possession of antiseptic qualities equal to those of any other substance; while used in moderation it is entirely free from smell, from unpleasant fumes, has no disagreeable taste, and is without any irritant or poisoning qualities. According to Professor Gamgee, by its use as an antiseptic, rawhide, meat, and other animal substances, immersed in a solution of 1.030 to 1.040, specific gravity, will be preserved perfectly for an indefinite period of time, and what is still more to the purpose, will not be attacked by insects after being removed from the solution. Fish, slightly tainted, when immersed recovers its freshness of appearance, and becomes firm and palatable. In some instances fresh fish, such as salmon, when caught were dipped in the solution, and after a passage of several days, without ice, to London, in the summer season, were found to be entirely eatable. This substance is suggested as an aid in drying cod on the coast of Newfoundland and elsewhere, as thereby an immense mass of fish that are now rejected could be readily preserved. The offal of cod and mackerel fisheries which is now thrown overboard, could be preserved by this substance as long as might be required, and then carried on shore to be converted into one or other of the various forms of fish guano.

For disinfecting purposes a solution varying from 1.006 to 1.010 is sufficiently strong to answer the desired object, stronger solutions being usually unnecessary and imparting a disagreeable smell. The solid matter of sewage is said to be precipitated more rapidly by this substance than by the use of the persalt of iron, and the odor disappears entirely. The use of chloralum in any epidemic, the cattle plague or other contagious disease, including the epizoötics, is indicated by the author of the communication. Finally, it is recommended for the treatment of wounds, erysipelas, gangrene, and various contagious and inflammatory diseases. It may also be used for the purpose of immersing the linen of patients before removing it from the sick chamber. For the purification of water-closets it is said to have no equal in any of the preparations hitherto recommended, and has also the advantage over nearly all the rest of being free from any offensive odor —*Agr. Report.*

***Pulsation of the Ureters.***

Dr. Asa Horr, of Dubuque, Iowa, writes to us of an interesting case, and asks whether it be unique or not. Will any of our readers answer the Doctor through our columns? He says that he has under treatment a case of vesico-vaginal fistula, where the cervix and os are hidden, and the orifice so distended by fibrous bands, that he can observe the discharge of the urine into the bladder, and states that it comes from the ureters in pulsations, with regular intervals of from fifteen to sixteen seconds, the pelves of the kidneys apparently contracting after the manner of the heart's action. To us the case is unique, and we shall be pleased to learn of a similar one on record.—*N. W. Journal.*

***Death from Chloral Hydrate.***

Dr. George G. Needham reports in the *Journal of Psychological Medicine* a case of fatal cerebral congestion following the administration of hydrate of chloral to a married woman, aged fifty, of hysterical diathesis, who had suffered for some two years with symptoms of mental derangement, consisting of distressing "nervousness," fear of impending death, hesitation, suspiciousness, etc. Ophthalmoscopic examination showed an enlarged and tortuous condition of the retinal vessels. In October, 1870, the loss of a relative threw her into a state of much excitement, for which she took, on October 19th, 115 grains of bromide of potassium. On the 21st, chloral hydrate was prescribed in thirty-grain doses, of which she took six, as follows: On the 21st, at 5.30 p.m. and 11 p.m.; on the 22d, at 10 a.m. and 3 p.m.; on the 23, at 1 a.m, 8.10 a.m., and 1.30 p.m. On the afternoon of the 22d she was sleeping quietly, with a somewhat rapid pulse, and was found in the same condition at two visits (morning and evening) on the 23d. On the morning of the 24th, her continued sleep created alarm, and ineffectual attempts were made to rouse her, which were maintained during the day and night. Sulphate of strychnia was thrice injected, in doses of one-thirtieth of a grain at intervals of four hours during the night. Coma progressed to a fatal termination on the afternoon of the 25th. The autopsy revealed extreme hyperæmia of the pia mater and brain substance. A year before, the patient had taken nearly the same quantity of chloral within the same period of time without ill effects. The writer suggests that the previous administration of a long course of bromide of potassium may increase the danger of full doses of chloral.—*Med. Gazette.*

### *Quinine in the Diseases of Childhood.*

C. Bing, M.D., Prof. of Pharmacology in the University of Bonn, Germany, (*Am. Jour. of Obs.*) regards quinine as an important remedy in those diseases of childhood dependent on septic or zymotic conditions, like measles, scarlatina, and diphtheria. In scarlet fever, quinine should be given from the *very commencement* in sufficiently large doses, the progress of the disease carefully watched by the aid of the thermometer, and the doses increased in quantity if the fever grows threatening.

Of the acute exanthemata of infants, he would mention one particularly as being within the sphere of the influence of quinia, namely, *erysipelas neonatorum.* As a general rule, an internal dyscrasia, or an external putrid ulceration of the navel, is assumed as the cause of this fatal disease.

The action of quinine in this disease is attributed to the overcoming of the alteration of the blood, to the diminution of the high temperature, and to the direct removal of the histological causes producing the erysipelas.

In pertussis, quinine has answered his expectations. Three conditions are absolutely necessary if we desire any good results from it in whooping cough. It should be given in solution; the dose should not be too small, and should not be administered in a vehicle that will prevent it from coming in contact with the mucous membrane in its passage through the pharynx. The preparation should only be given when dissolved in muriatic acid, unless we are desirous of employing the alkaloid combined with that salt.—*Med. Record.*

### *Sickness and Diarrhœa of Children.*

In this troublesome and frequent affection of children, a drop of ipecacuanha wine, administered every hour, is most successful. Under its influence the sickness almost immediately subsides, and the diarrhœa abates, although the latter may continue one or two days longer, and in a few cases, although very much controlled, may require another remedy to remove it. Its use is indicated when the motions are *frequent* and *slimy*, and also when they are of a *grass-green color;* and it is highly efficacious in this form of dysentery, when accompanied by vomiting; but the presence of sickness may be accepted as a special indication of its usefulness, and rarely will it be found to fail where sickness and slimy diarrhœa are present. The notes of numerous cases have been preserved, but it is unnecessary to give a detailed account of them, as they all present the symptoms above mentioned.—*C. C. Fuller, in* "*Lancet,*" Dec. 4, 1869.—*Nashville Jour. of Med. and Surg.*

### *Varying Effects of Poisons on Different Animals.*

It is a well-known fact that what is poisonous to one animal may be taken by another with entire impunity. In illustration of this proposition, we are informed that strychnine, so fatal to most animals, may be eaten by certain species of monkeys with perfect safety. In the case of an East India monkey, known as the Lungoor, (*Presbytis entellus*), one grain was first concealed in a piece of cucumber, which was eaten by the animal with no apparent effect. Three grains were afterward given, and with the same result. To test the strychnine used, three grains were administered to a dog, which proved almost immediately fatal. Another Indian monkey, known as the pouch cheek monkey, has been found to be more susceptible than the Lungoor, but not so much so as the dog.

It is also stated that pigeons can take opium in large quantities with no injurious consequence; goats, tobacco; and rabbits, belladonna, stramonium, and hyoscyamus.—*Agr. Report.*

### *Tetanus.*

Dr. David W. Yandell, of Louisville, Ky., (in the Am. Practitioner), lays down the following conclusions in regard to tetanus:

1. The traumatic tetanus occurs in males in the proportion of four to one, and tends to recovery oftenest in females.
2. That tetanus is most fatal in persons under ten years of age; that it is least fatal between ten and twenty years.
3. That traumatic tetanus usually supervenes between four and nine days after the injury, and these cases represent the largest mortality.
4. Recoveries from traumatic tetanus have been usually in cases in which the disease occurs subsequent to nine days after the injury.
5. When the symptoms last fourteen days, recovery is the rule, and death the exception, apparently independent of the treatment.
6. Of all the forms of tetanus, that appearing in the puerperal state is the most fatal.
7. That chloroform, up to this, has yielded the largest percentage of cures in acute tetanus.
8. The true test of a remedy for tetanus is its influence on the history of the disease. (1) Does it cure cases in which the disease has set in previous to the ninth day? (2) Does it fail in cases whose duration exceeds fourteen days?
9. That no agent, tried by these tests, has yet established its claim as a true remedy for tetanus.

### *Tin Foil as a Preservative Wrapping.*

Experiments conducted by E. Baudrimont with reference to the determination of the value of tin foil as a preservative wrapping for substances likely to deteriorate upon exposure to the air, have resulted in establishing its great usefulness. Boxes lined with it preserve botanical substances from deterioration a much longer time than boxes not so lined. Quick-lime wrapped in tin foil is prevented from slacking. Deliquescent salts are also kept from dissolving through the absorption of moisture from the air, when wrapped in this material. A large number of substances liable to change on exposure to the air were also protected in the same way. Some doubt having been expressed as to the impermeability of ordinary foil to air and moisture, these experiments will settle this question finally.—*Merc. Journal.*

### *Delirium Tremens.*

Dr. Murchison, in a clinical lecture at the Middlesex Hospital, has laid down some rules concerning the treatment of delirium tremens, which are of much practical value. The administration of alcohol he condemns as "only adding fuel to the fire," and maintaining congestion of the stomach, liver and kidneys; and he has long been in the practice of giving none, except in cases where there has been evidence of fatty heart, or an intermitting pulse, or some complication calling for its use. As much nutritious food as the patient can digest should of course be given. With regard to hypnotic drugs, he says: "Whenever the urine contains albumen as the result of recent congestion or old disease of the kidneys, opium is almost certain to fail, or even prove injurious; and, accordingly, it is a good rule never to give opium until an opportunity has been offered to test the urine. But when the urine has been ascertained to be free from albumen, opium may be given without fear, and usually with the best results" A full dose at first, followed by smaller ones every three hours until sleep ensues, is recommended. Digitalis, in doses of from fifteen to thirty minims of the tincture every four hours, is "particularly indicated in cases where the urine is scanty or contains albumen, or where the patient is very excited." Bromide of potassium he had not found of much service in securing sleep, though it may moderate active delirium or mental excitement. Hydrate of chloral is "particularly applicable in those cases where opium is contra-indicated. It does not, like opium, interfere with elimination by the kidneys; and, indeed, there are grounds for believing that the existing impurities of the blood favor the action of the chloral by assisting in the liberation of chloroform. One caution is necessary with regard to it. Not only in delirium tremens, but in other diseases, the first action of chloral (like that of an insufficient dose of chloroform) is exciting rather than sedative. You must not on that account infer that it is acting injuriously, for a second dose will often produce the desired sleep. The best way to give it is in doses of half a drachm every two or three hours until sleep results."—*Med. Gazette.—Med. Archives.*

### *Prepared Meat-Extracts in Java.*

It has frequently been remarked that the best inventions of the western nations have, in nearly every instance, been anticipated by processes long since devised and in use by the Orientals, especially by the natives of China and Japan; and we are assured that the subject of prepared meat-extracts takes its place in this category. We are informed by a recent communication of Dr. Pott, that the inhabitants of Java have for many years been in the habit of preparing flesh extracts of various kinds, and especially of beef, fish, and crabs, and that in this form they enter very largely into the internal commerce of the country. The preparation is known by the general name of *petis*, while the particular substance, whether the flesh of one of three kinds of oxen, of fish, or of crabs, is indicated by a special affix.

The preparation of the petis appears to be a very simple one, consisting merely in boiling the raw material and chopping it very fine, and then putting it in a press and forcing out all the juices. This juice is then boiled down at a moderate temperature to the consistency of syrup, and kept for use. As a general rule, the preparation is made of such pieces of meat of all the animals used as when brought to market are not sold before its close, a precaution rendered necessary by the heat of the country, and the impossibility of obtaining ice, by means of which to carry the food over until the next day. The substance from which the petis is expressed is also dried and introduced into commerce, but is generally used immediately, while the petis is distributed widely throughout the Indian Archipelago, and can be kept a long time. These preparations have an extremely saline taste, due almost entirely, however, to the concentration of the organic salts originally contained in the expressed juice. The smell is said to be quite agreeable, and the taste very appetizing.—*Agr. Report.*

### *Pepsin and its Medical Preparations.*

Dr. Fred. Hoffmann, of New York, relates, in a recent publication in the Proceedings of the American Pharmaceutical Association, a series of comparative examinations of the French as well as of the American commercial dry pepsins, by which he has satisfied himself of the well-founded disfavor into which this remedial agent has fallen with the practitioner. The author refers to similar results recently published by Dr. Hager, of Berlin (*Pharmac. Central Halle,* 1870), wherein Dr. Hager states that this remedy had been in discredit with him for years, but that his attention had been drawn to pepsin anew by the remarkable efficacy of Schering's recently introduced "Essence of Pepsin," prepared from dried ostrich's stomachs, at the advice of Dr. Oscar Liebreich. Dr. Hager examined, and gives in his paper the formulas of, the French liquid pepsin preparations. He found none of them equal in efficacy to Schering's preparation, which dissolves, at the temperature of the human body, one-tenth of its weight of humid blood-fibrin within one hour.

Dr. Hoffmann concludes his paper, "According to long experience and to the numerous and recent statements of eminent practitioners and observers, there remains no doubt that pepsin, when properly obtained and prepared, is of great therapeutical value in cases where the secretion of the gastric juice is either deficient in quantity or defective in quality, and that the discredit attributed to it is due to the inferior quality of the pepsin preparation, and to the defective mode of obtaining and preparing the same. It ought to be the aim, and lies in the self-interest of the pharmaceutist, henceforth to remedy the unreliability of this important preparation, and so furnish the practitioner and the patient with a pepsin in the most judicious form of preparation.

"In order to secure this desideratum I think it necessary to discard all amylaceous and similar so-called dry pepsin. Being a remedial agent of no chemical stability, and perhaps in a state of continuous self-decomposition when in contact with organic substances and with the atmosphere, our dry amylaceous pepsins are subjected to constant and incidental degradation. Sooner or later they will lose all medical power, and become totally inert. I believe that the best mode of obtaining and preserving the pepsic principle of the gastric juice of the stomach is its incorporation into inactive liquid menstrua without any chemical treatment, as is the case in Boudault's process, and in that of the French code. The simplest possible menstruum, perhaps, may be a mixture of glycerin and water, with or without an addition of some per centage of alcohol flavored with any artificial wine or fruit essence. The quantity of alcohol, however, ought not to exceed ten per cent., since a greater admixture probably neutralizes the therapeutic action of pepsin.

"The best mode of obtaining the gastric juice from the cleansed, fresh stomachs of a calf, pig, sheep, or of the turkey, is to squeeze them repeatedly between wooden rollers. After all the juice has been squeezed and collected with silver spoons, the membranes are each time, before again passing through the rollers, brushed with a mixture of equal parts of water and glycerin. By this operation, the membranes are thoroughly exhausted. According to our present experience, it may be best to add to the obtained juice a mixture consisting of twenty-five weight parts of glycerin, two parts of hydrochloric acid, eight parts of alcohol, and sixty-five parts of water, deprived of atmospheric admixture by previous boiling. But in order to make the preparation more agreeable to the taste I use, instead of the water, good Rhine wine, which by its simplicity and greater reliability as to its genuineness is greatly preferable to our commercial Madeira, sherry, and port wines. The mixture is then left in a very cool place (in summer in the ice-box), to deposit, in bottles entirely filled and well corked. When clear it is decanted, the rest filtered, and is then kept in a cool place in small bottles completely filled and tightly closed. The addition of hydrochloric acid may not be necessary, but it seems to be subservient to the clarification of the mixture.

"As to the strength of this pepsin wine, there is no standard established yet. I prepare it of the strength, suggested by Dr. Hager, to dissolve twelve and a half parts of its own weight of humid blood-fibrin."—*Medical Record.*

